# Enhanced Vascularization in Hybrid PCL/Gelatin Fibrous Scaffolds with Sustained Release of VEGF

**DOI:** 10.1155/2015/865076

**Published:** 2015-03-25

**Authors:** Kai Wang, Xuejiao Chen, Yiwa Pan, Yun Cui, Xin Zhou, Deling Kong, Qiang Zhao

**Affiliations:** State Key Laboratory of Medicinal Chemical Biology, Key Laboratory of Bioactive Materials, Ministry of Education, College of Life Sciences, Nankai University, Tianjin 300071, China

## Abstract

Creating a long-lasting and functional vasculature represents one of the most fundamental challenges in tissue engineering. VEGF has been widely accepted as a potent angiogenic factor involved in the early stages of blood vessel formation. In this study, fibrous scaffolds that consist of PCL and gelatin fibers were fabricated. The gelatin fibers were further functionalized by heparin immobilization, which provides binding sites for VEGF and thus enables the sustained release of VEGF. *In vitro* release test confirms the sustained releasing profile of VEGF, and stable release was observed over a time period of 25 days. *In vitro* cell assay indicates that VEGF release significantly promoted the proliferation of endothelial cells. More importantly, *in vivo* subcutaneous implantation reflects that vascularization has been effectively enhanced in the PCL/gelatin scaffolds compared with the PCL counterpart due to the sustained release of VEGF. Therefore, the heparinized PCL/gelatin scaffolds developed in this study may be a promising candidate for regeneration of complex tissues with sufficient vascularization.

## 1. Introduction

Blood vessels can provide necessary oxygen and nutrients to cell by diffusion processes, but the effective distance of diffusion is 100–200 *μ*m around a capillary [1]. Sufficient vascularization has a decisive effect on maintaining the survival of regenerated tissue in the field of tissue engineering. So far, only a few thin tissues, such as skin, cartilage, or cornea, can successfully survive after implantation [2]. Vascularization in three-dimensional tissue engineering scaffolds, such as bones, remained an important challenge in both research and clinic practice [3]. For the regeneration of blood vessels, immature and regressed capillaries within the wall of blood vessel also led to calcification after a long period of implantation, which resulted in the restenosis failure finally [4].

Recently, several approaches have been employed to improve vascularization of tissue engineering scaffolds, including optimization of 3D structures [5, 6]; preseeding of mesenchymal stem cells (MSCs) [7], endothelial cells (ECs) [2], and endothelial progenitor cells (EPCs) [8]; and the loading of bioactive molecules, such as NO donor [9], angiogenic factors [10, 11], or peptides [12] to the scaffold. However, until now none of them have been proven fully successful in development of long-lasting vasculature (blood vessels).

Rational design of physical structure of tissue engineering scaffold (pore size and interconnectivity) is fundamental for tissue regeneration and vascularization. Published results indicate that enhancing pore sizes within fibrous scaffolds fabricated by electrospinning can effectively promote cellular infiltration [13, 14]; thus, the vascularization has been improved thereafter. However, due to the bioinert characteristic of scaffolds, the integrity and stability of de novo vasculature are not satisfactory. More importantly, the capillaries will regress over long-term implantation as mentioned before.

In addition to the physical structure, bioactivity and function of the scaffold are more important for the vascularization. Of the known angiogenic factors, vascular endothelial growth factor (VEGF) is the most studied and effective one. It has been reported that loading VEGF onto tissue engineering scaffolds is an effective strategy to promote vascularization. However, due to the short half-life (about 50 min), high doses are often required, which results in a number of side effects (such as vasodilation and hypertension, inappropriate blood vessel growth, atherosclerotic plaque development, and neovascularization of tumors) [15, 16]. In addition, sustained local concentration of VEGF is also necessary for the development of mature blood vessels. Therefore, it is very important to develop functional fibrous scaffold that could load and release VEGF in a sustained and controlled manner.

Heparin possesses a special binding affinity to a variety of growth factors, including platelet-derived growth factor-BB (PDGF-BB) [17], fibroblast growth factor (FGF) [18], transforming growth factor-*β* (TGF-*β*) [19], and VEGF [20]. The binding affinity of heparin-binding growth factors with heparin is mainly based on electrostatic interactions [21], which is critical for storage, release, and protection of the growth factor from degradation [22]. For these reasons, heparin has been widely utilized as a functional molecule for the delivery of growth factors to achieve sustained releasing property.

In this study, a hybrid scaffold that consists of synthetic PCL fibers and native gelatin fibers was designed and prepared by coelectrospinning. Gelatin fiber was further crosslinked and functionalized by heparin in one step. Then VEGF was immobilized onto the heparinized PCL/Gel scaffolds by the aid of affinity interaction between heparin and VEGF. The sustained release of VEGF was confirmed by* in vitro* releasing test. The effect of released VEGF from the heparinized PCL/Gel scaffolds on enhancing vascularization has been investigated by both* in vitro* cell experiment and* in vivo* subcutaneous implantation.

## 2. Materials and Methods

### 2.1. Materials

Poly(*ε*-caprolactone) (PCL: *M*_*n*_ = 80,000), gelatin, and 1-ethyl-3-(3-dimethylaminopropyl) carbodiimide (EDC) were purchased from Sigma-Aldrich (Shanghai, China). 1,1,1,3,3,3-Fluoro-2-propanol (HFIP) was purchased from Tianli Technology Co. Ltd (Zhuhai, China). Heparin and 3-(4,5)-dimethylthiahiazo(-z-y1)-3,5-diphenytetrazoliumromide (MTT) were bought from Lianxing Biotechnology Company (Tianjin, China). 3,3′-Dioctadecyloxacarbocyanine perchlorate (DiO) and 1,1′-dioctadecyl-3,3,3′,3′-tetramethylindocarbocyanine perchlorate (DiI) were products of Molecular Probes (Eugene, OR). Human vascular endothelial growth factor (VEGF) was purchased from R&D Systems (Minneapolis, MN). Other reagents were purchased from Tianjin Sixth Reagent Company and used as received without further purification.

### 2.2. Electrospinning of PCL/Gel Fibrous Scaffolds

The hybrid fibrous scaffolds were prepared by coelectrospinning. A 25% w/v solution of PCL was prepared in a 5 : 1 (V/V) mixture of chloroform and methanol by stirring overnight. Gelatin was dissolved in the HFIP with stirring at room temperature for 6 h to obtain 6% w/v solution. Two 10-mL syringes were filled with PCL or gelatin solution and connected to a 21 G blunt-ended needle that served as the charged spinneret. The apparatus consists of a syringe pump (Cole Parmer, Vernon Hills, IL), a high-voltage generator (DWP503-1AC, Dong-Wen High Voltage power supply Factory, Tianjin, China), and a spinneret-mandrel as collector. The voltages between the needle tip and the rotating mandrel were set as 11 kV for PCL and 17 kV for gelatin. The distances between the needle tip and collector were 25 and 15 cm, respectively. The obtained electrospun scaffolds were vacuum-dried over 48 h at room temperature before further treatment.

### 2.3. Functionalization of PCL/Gel Fibrous Scaffolds

Crosslinking and heparization of electrospun PCL/Gel scaffolds were performed in 50% ethanol (v/v) containing 30 mM EDC and 0.5 mg/mL heparin for 12 h with gentle shaking in ice bath. The heparinized PCL/Gel scaffolds were washed 3 times with distilled water for 24 h in order to remove unreacted EDC and heparin completely. Then, the scaffolds were dried at room temperature before use.

The heparinized PCL/Gel scaffolds were cut into circular discs (1 cm in diameter) and placed in 48-well cell culture plate. Samples were sterilized by immersion in 75% v/v ethanol solution for 1 h and air-dried at room temperature. They were incubated in VEGF solution (100 ng/100 *μ*L in PBS) at 4°C overnight. Then, samples were quickly washed three times with fresh PBS.

### 2.4. Fiber Morphology and Distribution

The electrospun PCL/Gel scaffolds were mounted on an aluminum stubs and sputter coated with gold and palladium. They were observed by scanning electron microscope (SEM, HITACHI, X-650, Japan) at an accelerating voltage of 15 kV. Based on the SEM images, the pore diameter was determined according to the method described by Wang et al. [23]. At least six pores per image, three images per sample, and three samples per group were included to perform the calculation.

To visualize the distribution of the PCL and gelatin fibers, two different fluorescent dyes were incorporated into each fibers of the scaffold. DiI (1 mg/mL, orange-red fluorescent) was added to PCL solution and DiO (1 mg/mL, green fluorescent) was added to gelatin solution. After coelectrospinning, the PCL/Gel scaffolds were visualized using a laser scanning confocal microscope (CLSM; Zeiss LSM710).

### 2.5. Water Contact Angle (WCA) Measurements

The sessile drop method was used to measure WCA at room temperature through an optical contact goniometer (Harke-SPCA, Beijing, China). The scaffolds were pasted on glass slides and fixed onto the sample holder. Each measurement was performed using a 10 *μ*L drop of ddH_2_O on the surfaces of the scaffolds after 20 s. The average values of WCA were averaged based on four values at different positions of the sample surface.

### 2.6. Quantification of Immobilized Heparin

Circular samples (1 cm in diameter) of heparinized PCL/Gel were incubated with 5 mL of toluidine blue solution (0.04 g/100 mL 0.1 M HCl) for 4 h at room temperature with gentle agitation to form dye-heparin complex. Samples were rinsed with PBS twice for 5 min, and the residual dye that was bound to the heparin was solubilized with 5 mL mixed solution of ethanol and 0.1 M NaOH (4 : 1 v/v). The absorbance of the resulting solution was measured at 530 nm using a spectrophotometer, and the results were used to calculate the heparin concentration based on a calibration curve obtained from a series of graded dye-heparin complexes. The final heparin amount was normalized to the dry weight of each sample (*n* = 3).

### 2.7. Mechanical Test

Mechanical properties were measured on a tensile-testing machine with a load capacity of 100 N (Instron-5865, Norwood, MA). The 1 cm × 4 cm electrospun PCL/Gel scaffolds were clamped and pulled longitudinally at a rate of 10 mm/min. The tensile strength and elongation at break were measured. The Young's modulus was obtained by measuring the slope of the stress-strain curve in the elastic region.

### 2.8. VEGF Release

The VEGF loaded specimens were placed in 5 mL centrifugal tube, and 2 mL of release media (0.1% sodium azide in PBS) was added to each tube. They were maintained at 37°C with gentle shaking for up to 25 days. At predetermined time points, buffer in each tube was collected and replaced with fresh PBS. The amount of released VEGF was analyzed using a human VEGF ELISA kit according to the manufacturer's instructions. The cumulative amount of VEGF released from each scaffold was normalized to the dry weight of each sample (*n* = 3).

### 2.9. Cell Proliferation

Specimens were cut into circular discs (1 cm in diameter) and then placed in 48-well plate. Human umbilical vein endothelial cells (HUVECs) (ScienCell, USA) were seeded on the scaffolds at a density of 5.0 × 10^3^ cells/well. The endothelial culture medium (ECM, ScienCell, USA) was changed every 24 h. After cell culture for 1, 3, and 5 days, 50 *μ*L of MTT reagents (5 mg/mL in PBS) was added to each well and incubated for 4 h. Then, the supernatant was discarded and the cell-scaffold constructs were washed with PBS. 300 *μ*L of DMSO was added, and the plate was placed on a shaker for 15 min to dissolve the formazan salts. Finally, 100 *μ*L of dissolved solution from each well was transferred to a 96-well plate. The absorbance at 490 nm was measured with a Bio-Rad Microplate Reader (iMark, Bio-Rad, USA).

### 2.10. Subcutaneous Implantation in Rats

Sprague Dawley (SD) rats (male, weight 250–300 g) were purchased from the laboratory animal center of the Academy of Military Medical Sciences (Beijing, China). All animal experiments were approved by the Animal Care and Use Committee of Nankai University. The rats were anesthetized with intraperitoneal injection of chloral hydrate (0.3 mg/kg body weight). Three circular specimens (1 cm in diameter) were implanted subcutaneously at one side of the backbone. The grouping of animals was based on the type of scaffolds and duration of observation for 2 and 4 weeks.

### 2.11. Histological Analysis

Upon explantation, the surrounding tissues of the scaffolds were excised together and then fixed with 4% paraformaldehyde at 4°C overnight, dehydrated in a 30% sucrose solution for 24 h, and finally embedded in optimal cutting temperature compound (OCT) for sectioning. One part of sections was stained by H&E to assess cellularization. The cell migration rate was calculated by the following equation:

(1) $$\begin{array}{c} \text{The}\;\text{cell}\;\text{migration}\;\text{rate}=\frac{W}{W_{0}}\%, \end{array}$$

where *W* is the area of the cell migration into scaffolds and *W*_0_ is the area of the scaffold. The other sections were stained by immunofluorescence to assess vascularization. Monoclonal antibody to vWF was used as primary antibodies, and Alexa Fluor 488-conjugated anti-rabbit IgG (Invitrogen) was used as the secondary antibody. The nuclei were counterstained with DAPI containing mounting solution (Dapi-Fluoromount-G, Southern Biotech, England). The sections without incubation with primary antibody were used as negative controls. Slides were observed under a fluorescence microscope (Zeiss Axio Imager Z1, Germany) and a digital camera (AxioCam MRm, Germany). The mean blood vessel number was determined based on the fluorescence images. At least six images per section, four sections per sample, and three samples per group were included to obtain the calculation.

### 2.12. Statistical Analysis

All data were presented as means ± standard deviations. A two-tailed paired Student's *t*-test was used to compare the differences. A value of *P* < 0.05 was considered statistically significant.

## 3. Results and Discussion

### 3.1. Characterization of the Structure and Composition of the Scaffolds

In this study, PCL/gelatin scaffolds with hybrid fibrous structure were designed and fabricated by coelectrospinning. Of the two fiber components, synthetic polymer PCL provides mechanical support, while native polymer gelatin contributes the functional groups for further heparization and VEGF loading. By controlling the flow rate of PCL solution (Table 1), two types of PCL/Gel scaffolds with different compositons were prepared.

**Figure 1:**
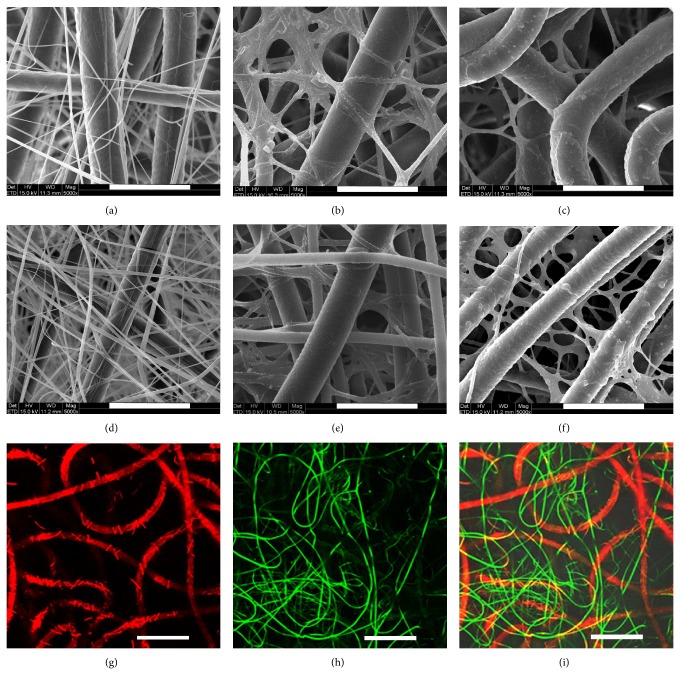
Structure characterization of electrospun PCL/Gel scaffolds. SEM images of PCL/Gel-1 (a), Hep-PCL/Gel-1 (b), and Hep-PCL/Gel-1 degraded in PBS for 14 days (c) and PCL/Gel-2 (d), Hep-PCL/Gel-2 (e), and Hep-PCL/Gel-2 degraded in PBS for 14 days (f) (scale bar = 20 *μ*m). Fluorescent images of PCL/Gel-1 with red PCL fibers labled with DiI (g), and green gelatin fibers labled with DiO (h), as well as the merge one (i) (scale bar = 20 *μ*m).

SEM images show two types of fibers with contrast in fiber size (Figures 1(a) and 1(d)). The fibers of large diameter are attributed to PCL, while those of small diameter correspond to gelatin, by comparing with the SEM images of neat PCL and gelatin [24]. The pore size of scaffolds was also determined based on the SEM images (Table 2).

In order to better discern the fibers distribution, two types of fibers were fluorescently stained, respectively. As shown in Figure 1(i), red PCL and green gelatin fibers were distributed randomly and uniformly. The relative ratio of two fiber componets was quantified through selective leaching of gelatin. Results indicate that the gelatin ratio was 6.87 wt% and 19.00 wt% for PCL/Gel-1 and PCL/Gel-2, respectively, which agrees well with the theoretical value determined according to the feeding ratio [25].

The electrospun scaffolds were further functionalized by immobilizing heparin under the catalysis of zero-length crosslinking agent, 1-ethyl-3-(3-dimethylaminopropyl) carbodiimide (EDC). Within this process, gelatin component was also crosslinked, which is necessary for gelatin fibers to maintain the stable structure [20, 26, 27]. After the functionalization, PCL fibers remained the well-fined morphology, whereas the gelatin fibers showed some degree of collapse and deformation, but fiber morphology could still be identified (Figures 1(b) and 1(e)). The average pore size did not show detectable change before and after the functionalization (Table 2).

The stability of heparinized PCL/Gel scaffolds was evaluated by* in vitro* degradation for 14 days. Significant degradation of gelatin fibers could be identified based on the SEM images (Figures 1(c) and 1(f)), which results in enhanced pore size of the scaffold (Table 2).

The heparization was quantified by Toluidine blue assay, and results indicate that the amount of immobilized heparin was consistent with the gelatin content (Table 1).

### 3.2. Surface Hydrophobic/Hydrophilic Property

**Figure 2:**
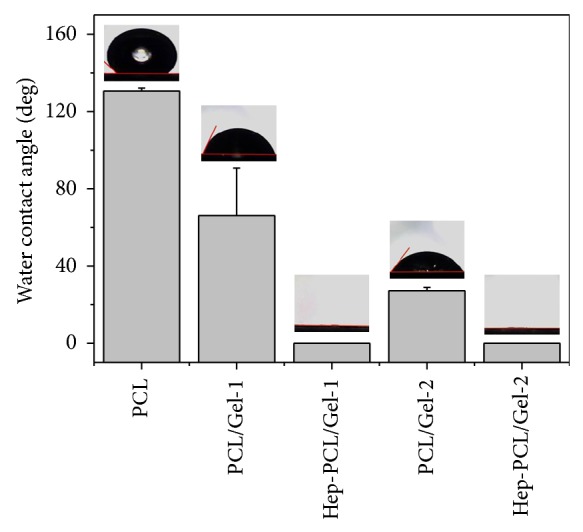
Surface hydrophilic/hydrophobic performance analyzed by water contact angle analysis (*n* = 5) and the corresponding images of water droplets on the different surfaces after contact of 20 seconds.

Surface hydrophilicity was analyzed by static contact angle measurement. In general, PCL is a hydrophobic polymer with water contact angle (WCA) of 130.52 ± 1.56°. After the incorporation of gelatin component, the hydrophicity was improved; that is, the mean WCA of PCL/Gel-1 and PCL/Gel-2 decreased to 70.85 ± 1.93° and 58.18 ± 1.73°, respectively. Heparization further enhanced the surface hydrophilicity, and PCL/Gel-1 and PCL/Gel-2 became fully hydrophilic; that is, the drop was completely absorbed within 20 seconds (Figure 2).

### 3.3. Mechanical Properties

**Figure 3:**
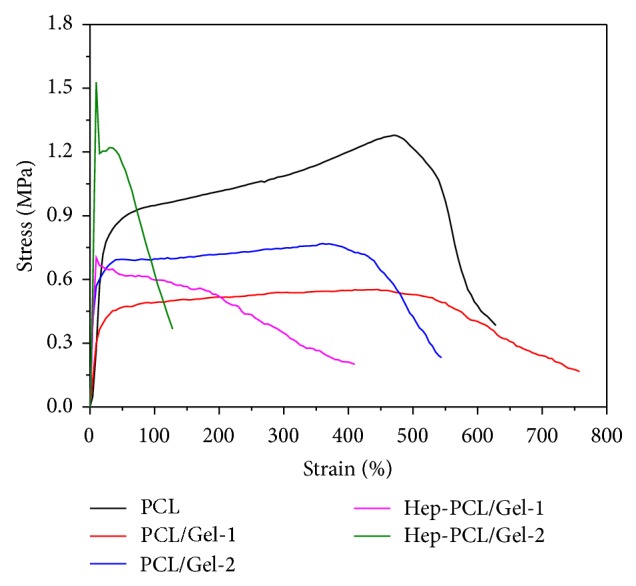
Mechanical properties of PCL and PCL/Gel scaffolds with or without heparization.

The mechanical properties were evaluated by tensile test (Figure 3 and Table 3). Neat PCL is a ductile polymer with high degree of elongation, that is, 614.90 ± 13.34%. The incorporation of gelatin component fails to alter the flexible mechanical characteristic; only tensile strength was slightly lowered because the weight ratio of the gelatin in the scaffold is relatively low. Heparization strengthens the PCL/Gel scaffolds effectively with evidently enhanced tensile strength and Young's modulus. At the same time, the elongation at break was adversely decreased due to the formation of 3D molecular network during the crosslinking. Similar trends in mechanical properties have been reported before [21]. However in this case the elongation for heparinized PCL/Gel is still higher than 100%, which is higher than the normal displacement of the artery during the physiological dilation and constriction, and satisfies the requirement of artificial vascular grafts. In a word, the mechanical properties of the scaffolds could be readily tuned within a wide range to satisfy the requirement of vascular grafts.

### 3.4. *In Vitro* Release of VEGF

**Figure 4:**
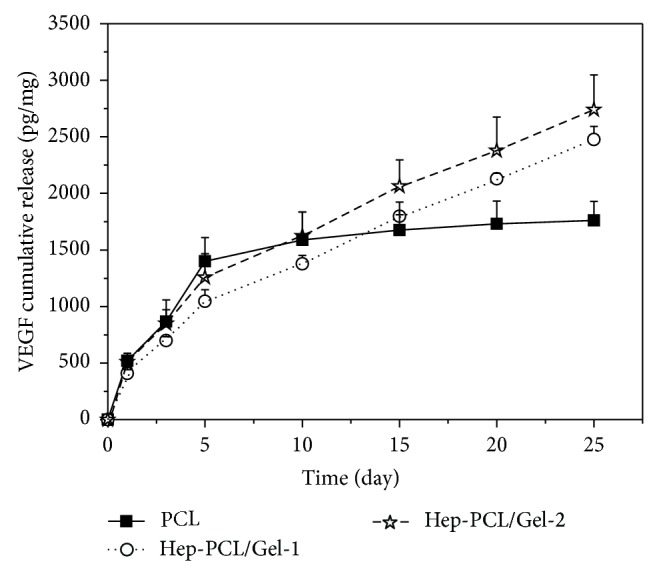
*In vitro* release of VEGF from heparinized PCL/Gel scaffolds and PCL (*n* = 3).

Figure 4 shows* in vitro* releasing profile of VEGF from heparinized PCL/Gel scaffolds with neat PCL as control. Sustained release of VEGF was observed in the Hep-PCL/Gel scaffolds within time period of 25 days. The release rate is quite stable, and the cumulative amounts of VEGF approach 2.48 ± 0.11 ng/mg for Hep-PCL/Gel-1 and 2.7 ± 0.31 ng/mg for Hep-PCL/Gel-2, respectively. In contrast, PCL shows burst release during the initial 5 days due to the passive physical adsorption of VEGF on the scaffold, and nearly no additional release occurred within the following time period.

The sustained releasing behavior could be attributed to the stabilization effect provided by the heparin. The high binding affinity between VEGF and heparin could effectively delay the dissociation of VEGF from the scaffolds; thus, sustained release of VEGF has been realized.

Heparin also demonstrates high affinity towards a wide range of cytokines and growth factors; therefore, it has been widely utilized for the construction of biomaterial-based delivery system [20, 28–30]. The specific interaction also effectively protects growth factors from thermal denaturation, enzymatic degradation, and inactivation at acidic pH, which mainly comes from the conformational change in the growth factor molecule during the binding with heparin [31, 32]. In addition, it has been reported that heparin also shows binding affinity towards some cytokines secreted by the inflammatory response, which has a positive effect on inhibiting inflammatory reaction [33].

During the implantation of blood-contacting materials or devices in clinic, heparin was often administrated with the aim of anticoagulation. However, the half time of exogeneously supplied free heparin is short (less than 2.5 hours) [34]. They will be eliminated from the body rapidly [35]; hence, the presence of them cannot alter the releasing kinetics of VEGF from heparinized PCL/Gel scaffolds markedly.

### 3.5. *In Vitro* Endothelial Cell Proliferation

**Figure 5:**
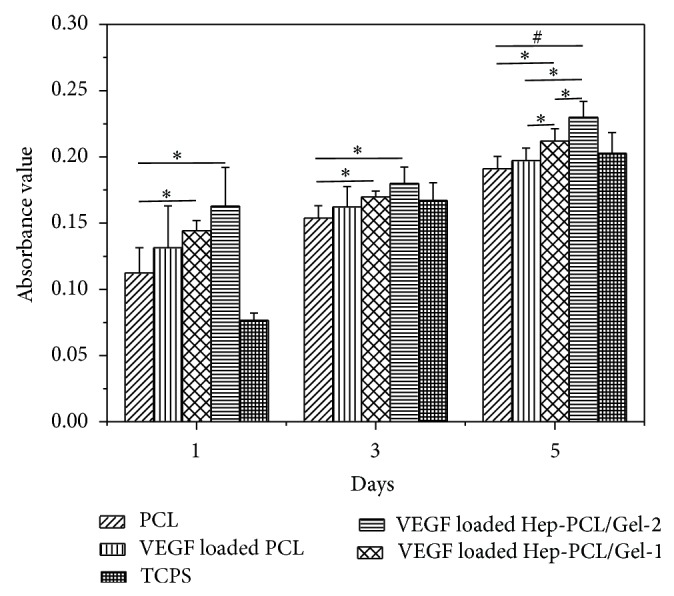
MTT assay for cell proliferation of human umbilical vein endothelial cells (HUVECs) on the VEGF loaded PCL and heparinized PCL/Gel scaffolds. Neat PCL and tissue culture plate (TCP) were used as control (*n* = 5). ^*^ *P* < 0.05; ^#^ *P* < 0.001.

The effect of sustained release of VEGF on the endothelial cell proliferation was investigated by* in vitro* MTT assay. As shown in Figure 5, the cell proliferation was significantly (*P* < 0.05) accelerated on VEGF loaded Hep-PCL/Gel scaffolds compared with that on the neat PCL throughout the entire culture process. In addition, cells grew faster on the VEGF loaded Hep-PCL/Gel-2 than on the VEGF loaded Hep-PCL/Gel-1 scaffold, which may be due to the higher VEGF loading. VEGF loaded on the PCL scaffold slightly increases the cell proliferation, but the effect is not pronounced. The enhanced proliferation on the VEGF loaded Hep-PCL/Gel scaffolds also confirms that the bioactivity of VEGF released from the scaffolds was higher than PCL scaffolds due to the protective effect provided by immobilized heparin.

### 3.6. *In Vivo* Angiogenesis

**Figure 6:**
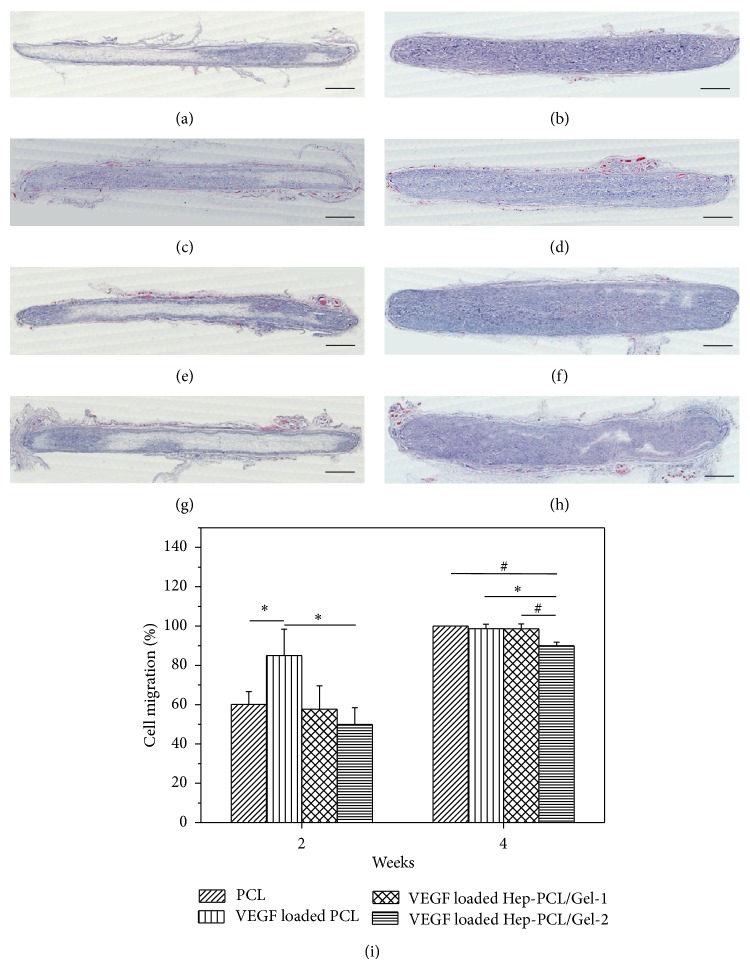
Representative hematoxylin and eosin (H&E) staining of explanted scaffolds after subcutaneous implantation for 2 (a, c, e, and g) and 4 (b, d, f, and h) weeks (*n* = 3): PCL (a and b); VEGF loaded PCL (c and d); VEGF loaded Hep-PCL/Gel-1 (e and f); VEGF loaded Hep-PCL/Gel-2 (g and h) (scale bars = 1 mm), and the corresponding quantitative analysis of cell migration (i). ^*^ *P* < 0.05; ^#^ *P* < 0.001.

Angiogenesis in the VEGF loaded PCL/Gel scaffolds was evaluated by subcutaneous implantation in rats. After 4 weeks of implantation, the H&E images clearly show that the scaffold was almost fully cellularized (Figures 6(b), 6(d), 6(f), and 6(h)). As mentioned before, rapid and sufficient cellularization is the prerequisite for vascularization. Previous studies have shown that the relatively small pore size in electrospun scaffolds often limits the cell infiltration. In this study, the pore size was optimized by the method previously developed [14], making the scaffold favorable for cell infiltration and migration. The cell migration rates in all groups exceed 90% after 4 weeks (Figure 6(i)).

**Figure 7:**
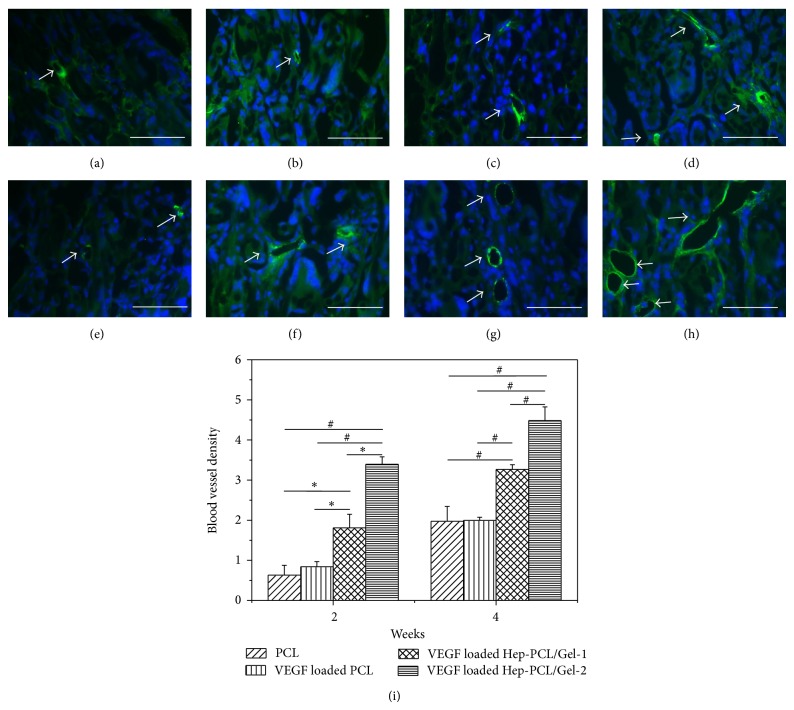
Representative microscopic images showing the blood vessels immunostained by vWF in the explanted scaffolds after subcutaneous implantation for 2 (a–d) and 4 (e–h) weeks (*n* = 3): PCL (a and e); VEGF loaded PCL (b and f); VEGF loaded Hep-PCL/Gel-1 (c and g); VEGF loaded Hep-PCL/Gel-2 (d and h) (scale bars = 50*μ*m). Quantitative analysis on the density of blood vessel (i). ^*^ *P* < 0.05, ^#^ *P* < 0.001.

The blood vessels in the cellularized area were analyzed by immunofluorescent staining with vWF (Figures 7(a)–7(h)) and further quantified based on the image. More capillaries could be clearly identified on the VEGF loaded PCL/Gel scaffolds (arrow indicated). The quantitative result further demonstrates that VEGF loaded PCL/Gel scaffolds significantly enhanced the vessel density compared to PCL and VEGF loaded PCL (*P* < 0.001) after 4 weeks. This enhancement is closely associated with heparin for VEGF loading and protecting, which agrees well with* in vitro* cell proliferation (Figure 5).

Recently, numerous strategies have been developed to deliver VEGF in tissue engineering scaffolds in order to promote angiogenesis. Among these approaches, covalent binding is a robust one, achieving stable binding of VEGF [36]. However, it involves multiple steps, which is tedious and may compromise bioactivity of the protein [37]. The physical absorption by polymer-based carrier is a convenient and effective alternative [38]. This strategy can achieve sustained release of VEGF to some extent [37], but the simple adsorption fails to provide protection for VEGF. In contrast, the affinity delivery of VEGF based on the heparinized scaffold can not only effectively release VEGF in a controlled manner but also can protect it from degradation. The heparin conjugated PCL/Gel scaffolds therefore demonstrated excellent vascularization* in vivo*.

## 4. Conclusions

In summary, a type of tissue scaffold with hybrid fibrous structure was successfully prepared in this study using two fiber components. Synthetic PCL provides optimal mechanical strength, and native polymer gelatin has been further heparinized in order to deliver VEGF in a controlled manner. The scaffolds show well-defined fiber morphology and homogeneous distribution before and after heparin-functionalization. The physical properties of as-prepared scaffolds, including mechanical properties and surface hydrophilicity, have been evaluated and satisfy the requirement of tissue engineering scaffolds. Heparinized scaffolds demonstrate sustained release of VEGF, which proceeded beyond a time period of 25 days. The sustained release of VEGF can evidently promote the vascularization, which has been confirmed by both* in vitro* cell proliferation and* in vivo* subcutaneous implantation assay.

## Figures and Tables

**Table 1:** Preparation of heparinized PCL/Gel fibrous scaffolds.

Sample code	*C*_PCL_ (g/mL)	*C*_Gel_ (g/mL)	Flow rate PCL (mL/h)	Flow rate Gel (mL/h)	Immobilized heparin (*µ*g/cm^2^)
PCL	0.25	0	8	0	0
PCL/Gel-1	0.25	0.06	8	4	0
PCL/Gel-2	0.25	0.06	4	4	0
Hep-PCL/Gel-1	0.25	0.06	8	4	39.28 ± 4.24
Hep-PCL/Gel-2	0.25	0.06	4	4	84.11 ± 9.24

**Table 2:** The average pore sizes of the scaffolds.

Scaffolds	Averaged pore size (*µ*m)
PCL	37.61 ± 12.19
PCL/Gel-1	12.43 ± 5.96
PCL/Gel-2	8.07 ± 3.31
Hep-PCL/Gel-1	13.37 ± 6.46
Hep-PCL/Gel-2	8.46 ± 4.24
Hep-PCL/Gel-1 in PBS for 14 d	26.94 ± 14.76
Hep-PCL/Gel-2 in PBS for 14 d	20.94 ± 16.57

**Table 3:** Mechanical properties of the scaffolds (*n* = 3).

Sample code	Tensile strength (MPa)	Young's modulus (MPa)	Elongation at break (%)
PCL	1.18 ± 0.14	4.41 ± 2.05	614.90 ± 13.34
PCL/Gel-1	0.55 ± 0.04	3.02 ± 0.04	741.78 ± 35.12
PCL/Gel-2	0.76 ± 0.01	5.85 ± 0.01	551.37 ± 10.56
Hep-PCL/Gel-1	0.73 ± 0.02	7.57 ± 0.20	394.52 ± 29.36
Hep-PCL/Gel-2	1.42 ± 0.11	14.80 ± 1.47	126.69 ± 15.18
